# Intranasal, siRNA Delivery to the Brain by TAT/MGF Tagged PEGylated Chitosan Nanoparticles

**DOI:** 10.1155/2013/812387

**Published:** 2013-09-12

**Authors:** Meenakshi Malhotra, Catherine Tomaro-Duchesneau, Shyamali Saha, Satya Prakash

**Affiliations:** ^1^Biomedical Technology and Cell Therapy Research Laboratory, Departments of Biomedical Engineering, Faculty of Medicine, McGill University, 3775 University Street, Room 311, Lyman Duff Medical Building, Montreal, QC, Canada H3A 2B4; ^2^Faculty of Dentistry, McGill University, 3775 University Street, Montreal, QC, Canada H3A 2B2

## Abstract

Neurodegeneration is characterized by progressive loss of structure and function of neurons. Several therapeutic methods and drugs are available to alleviate the symptoms of these diseases. The currently used delivery strategies such as implantation of catheters, intracarotid infusions, surgeries, and chemotherapies are invasive in nature and pose a greater risk of postsurgical complications, which can have fatal side effects. The current study utilizes a peptide (TAT and MGF) tagged PEGylated chitosan nanoparticle formulation for siRNA delivery, administered intranasally, which can bypass the blood brain barrier. The study investigates the optimal dose, duration, biodistribution, and toxicity, of the nanoparticle-siRNA formulation, in-vivo. The results indicate that 0.5 mg/kg of siRNA is delivered successfully to the hippocampus, thalamus, hypothalamus, and Purkinje cells in the cerebellum after 4 hrs of post intranasal delivery. The results indicate maximum delivery to the brain in comparison to other tissues with no cellular toxic effects. This study shows the potential of peptide-tagged PEGylated chitosan nanoparticles to be delivered intranasally and target brain tissue for the treatment of neurological disorders.

## 1. Introduction 

Blood brain barrier (BBB) is the major challenge that limits the application of neurotherapeutics for the treatment of neurological disorders [1]. The BBB is formed by a membranous network of brain capillary endothelial cells (BCEC), connected through tight junctions [2]. This physiological barrier imposes a selective permeability to various molecules and substances. This close-knit microenvironment is however essential to protect central nervous system (CNS) from the intrusion of harmful chemical/substances, but it poses a challenge for the delivery of neuroprotective drugs for the treatment of neurological disorders [3]. Systemic administration of various neuropeptides and hydrophilic therapeutic agents, such as antibiotics and anticancer agents, has failed to cross the BBB [4]. The CNS only allows small, lipophilic compounds (<400–500 Da) to permeate and cross the BBB [1]. The cerebrovasculature of CNS has a large surface area of approximately 20 m^2^, which allows successful drug administration *via *transendothelial route, provided that the physiological barrier could be overcome. Current clinical strategies include surgical interventions, which are invasive and can later pose postsurgical complications with fatal side effects [5]. Some of the currently employed invasive approaches (mechanically breaching the BBB) include (a) interstitial delivery [6], intracerebroventricular delivery [7], intracerebral delivery [8], and convection enhanced delivery [9].

In contrast to invasive strategies, intranasal delivery is emerging as a noninvasive approach to deliver therapeutics to the brain, bypassing the BBB [10–13]. Intranasal delivery has shown success of delivering peptides and growth factors to CNS, over intravenous delivery [14, 15]. Moreover, unlike parenteral route, intranasally administered drugs avoid elimination by liver, kidney filtration, gastrointestinal tract, and serum degradation [16]. The passage across the nasal epithelium is suggested to be a transcellular route for high molecular weight molecules, such as proteins, peptides, and nucleic acids [16]. In general, the small molecules follow a paracellular path through tight junctions between the cells in the nasal epithelium, and the other molecules follow endocytic pathways, such as receptor mediated transport mechanism. [16]. Most of the intranasally administered drugs have shown <0.1% of success to be transported directly to the brain, but various drug delivery formulations, such as nanoparticles, have shown to enhance the drug permeability across the olfactory epithelium [17]. However, the exact pathway or responsible features (nanoparticles size and nasal surface area) that underlie the selective transport of nanoparticles through olfactory epithelial cells to the brain are yet to be fully elucidated [18].

RNA interference (RNAi) is emerging as a class of therapeutic drug that offers specific silencing of the targeted gene at mRNA level, leading to inhibition of protein synthesis [19]. It is mediated by double stranded short interfering RNAs (ds siRNA), which are approximately 13 KDa in molecular weight and 18–22 bp in length [19]. Successful delivery of siRNA has been a challenge due to its transient nature. In-vivo, siRNAs are susceptible to enzymatic degradation, low cellular uptake, rapid clearance from the blood, and off-target effects [20]. Although various chemical modifications and use of transfection agents have successfully combated the stability and cellular uptake issues of siRNAs, the challenge lies in the clinical application of siRNA for CNS delivery [21]. In recent years, there have been important advances in the field of nanotechnology, and nanoparticles such as polyplexes, dendriplexes, and exosomes have shown success with regards to the delivery of siRNA, in-vivo [22–24]. Chitosan, a polycationic polymer, has been widely used to deliver various therapeutics including nerve growth factors, insulin, and drugs to the brain via intranasal route of delivery [25–27]. Chitosan is known to be a mucoadhesive agent; the amines in chitosan react with sialic residues present on the mucosal layer that helps reduce clearance rate from nasal cavity [28]. Due to its mucoadhesive property, it has been used for intranasal delivery of various formulations for ocular and pulmonary diseases [29–33]. In the present study, we have utilized the ability of surface functionalized chitosan nanoparticles to deliver siRNA to the brain, following an intranasal route. The study is a qualitative investigation of the siRNA delivery through the surface functionalized chitosan nanoparticles. The study determines the optimal dose of siRNA-nanoparticle formulation for delivery to the brain (cerebral cortex and cerebellum). Biodistribution and local toxicity of the formulation in different organ tissues are also investigated.

## 2. Materials and Methods

### 2.1. Materials

Low molecular weight (LMW), 10 KDa chitosan (CS) was obtained from Wako (Richmond, VA, USA), having a viscosity of 5~20 cP and a degree of deacetylation of 80.0%; polyethylene glycol monomethyl ether (mPEG) (M.W. 2,000), sodium tripolyphosphate (TPP), and glacial acetic acid of analytical grade were obtained from Sigma (Oakville, ON, Canada). Trans-activated transcription (TAT) peptide (NH_2_-RKKRRQRRR) M.W. 1339.63 and mechano growth factor (MGF) peptide (YQPPSTNKNTKSQRRKGSTFEEHK-NH_2_) M.W. 2848.14 were synthesized by Sheldon Biotech, McGill University. Biotin-tagged scrambled siRNA, siGENOME Nontargeting siRNA #2: D-001210-02, was procured from Dharmacon Inc. (Lafayette, CO, USA). 

### 2.2. Preparation of siRNA-Nanoparticle Formulation

The nanoparticles were prepared from a synthesized peptide-tagged PEGylated chitosan polymer. The peptides used in this study were MGF and TAT. The nanoparticles were synthesized as described previously [34, 35]. In brief, the derivatized polymer CS-PEG-TAT/MGF was dissolved in 1% acetic acid solution (0.5 mg/mL) at pH 5.0. The polymer was heated at 60°C and sonicated to ensure maximum dissolution. The polymer was filtered using 0.8 *μ*m filter before forming nanoparticles. TPP at 0.7 mg/mL, pH 3.0, was used as a crosslinker to form nanoparticles. The biotin-tagged scrambled siRNA (2 *μ*g) was premixed with 200 *μ*L of TPP and dropped into the 800 *μ*L of CS-PEG-TAT/MGF polymer solution, under a constant magnetic stirring at 800 rpm for an hour. CS-PEG-TAT/MGF nanoparticle formulations complexing scrambled biotin-siRNA were concentrated to 4 different doses: (a) 0.25 mg/kg, (b) 0.5 mg/kg, (c) 1 mg/kg, and (d) 2 mg/kg of animal weight, using Amicon Ultra-15 centrifugal filters (molecular weight (MW) cut-off 3000 Daltons, Millipore). The morphology and size of the nanoparticles were observed under transmission electron microscopy (TEM). 

### 2.3. Animals

Four-week old C57BL/6J male mice, weighing 10–15 g, were purchased from MMRC facility in Jackson Laboratory (Bar Harbor, ME, USA). The animals were housed in an environment with controlled temperature (22°C), humidity, and a 12 h light/dark cycle at McGill's animal care facility. The animal experiment was conducted as per the protocol approved by the animal care committee at McGill University (Montreal, QC, Canada). Standard mouse chow and water were supplied ad libitum. Animals were acclimatized for a week before the experiment. 

### 2.4. Intranasal Nanoparticle-siRNA Delivery

The animals were block randomized into 5 groups with *n* = 2 in each group to receive different concentrations of scrambled biotin-siRNA complexed with CS-PEG-TAT/MGF nanoparticles (0.25 mg/kg, 0.50 mg/kg, 1 mg/kg, 2 mg/kg, and PBS as control). The animals were anesthetized with a 75–100 *μ*L cocktail comprising ketamine (100 mg/kg), xylazine (10 mg/kg), and acepromazine (3 mg/kg) via intraperitoneal administration. The animals were placed in a head back position after anesthesia to deliver nanoparticle-siRNA formulations. A total of 30 *μ*L of the nanoparticle-siRNA formulation was administered intranasally once (5 *μ*L/drop) over 15–20 minutes. The experimental end points were 4, 16, 24, and 48 h. 

### 2.5. Histology

The animals were anesthetized using the aforementioned cocktail and perfusion fixed with 4% paraformaldehyde (PFA) (Sigma Aldrich, Canada) at each end point. Brain, lungs, heart, stomach, kidney, and liver were harvested and kept at 4°C in 4% PFA for 48 hrs. The tissues were trimmed to 3 mm thick sections and stored in 70% ethanol in histology cassettes. The tissues were paraffin-embedded and processed into 4 *μ*m thick section on slides (The Rosalind and Morris Goodman Cancer Research Centre, McGill University). The tissue slides were stained with Vectastain elite ABC kit (Vector laboratories; Burlingame, CA, USA) as per the manufacturer's protocol and diaminobenzidine (DAB) was used as a substrate to assess the presence of biotin tag present on siRNA (brown staining). Hematoxylin was used as a counterstain and slides were mounted with permount (Vector laboratories; Burlingame, CA, USA) and observed by compound microscopy (Leica DM500; ON, Canada) at 400x.

### 2.6. Toxicity Analysis

Analysis of apoptotic cells was performed using terminal deoxynucleotidyl transferase-mediated dUTP nick-end labeling (TUNEL) staining (Promega, Madison, Wisc., USA) as per the manufacturer's protocol after blocking biotin-siRNA. The tissue sections in paraffin block were dewaxed in xylene, rehydrated in decreasing concentrations of ethanol, and washed with PBS. The tissue sections were then incubated with streptavidin-HRP reagent (Roche Diagnostics) for 10 min and washed in PBS. Then, the sections were incubated with 3% H_2_O_2_ for another 10 min and washed in PBS. The rest of the TUNEL assay was performed as per the manufacturer's instructions. The tissue sections were counter stained with hematoxylin, washed in distilled water, dehydrated and mounted with permount (Vector laboratories; Burlingame, CA, USA), and observed by compound microscopy at 400x.

### 2.7. Statistical Analysis

Experimental results are expressed as means ± standard error of the mean (SEM). Statistical analysis was carried out using SPSS Version 17.0 (Statistical Product and Service Solutions, IBM Corporation, New York, USA). Two-sided statistical comparisons were carried out using the general linear model and Tukey's post hoc analysis, assuming equal variances, independence, and normality. Statistical significance was set at *P* < 0.05, and *P* values less than 0.01 were considered highly significant.

## 3. Results

### 3.1. Characterization of Chitosan-PEG-Peptide

**Figure 1:**
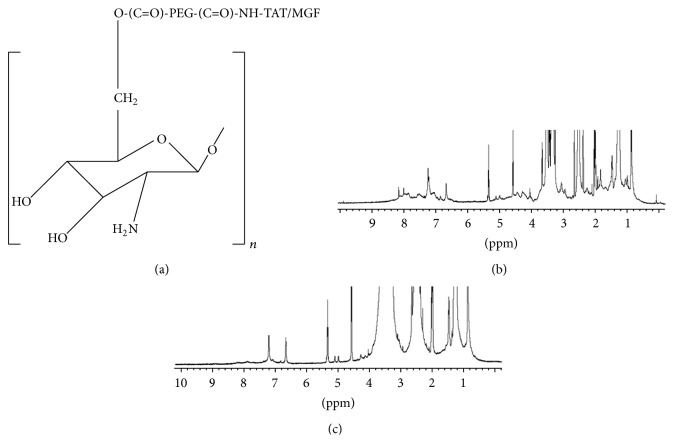
(a) Chemical structure of chitosan-PEG-TAT/MGF polymer, ^1^H NMR spectra of (b) chitosan-PEG-MGF (CS-O-PEG-CONH-MGF), and (c) chitosan-PEG-TAT (CS-O-PEG-CONH-TAT). The multiple peaks of oxymethyl groups in PEG at *δ* 3.3 to 3.7 cover over the signals of pyranose ring of chitosan. The multiple peaks at *δ* 6.5–8.5 belong to the peptide MGF and *δ* 6.5–8.5 belong to the TAT peptide sequence, respectively.

The peptide-tagged PEGylated chitosan polymer was synthesized following a series of chemical reactions as previously published by our group [34].  Figure 1(a) represents the final chemical structure of the synthesized peptide-tagged PEGylated chitosan polymer and their respective proton nuclear magnetic resonance spectroscopy (^1^H NMR) spectras (Figures 1(b) and 1(c)). As represented in Figures 1(b) and 1(c), the multiple peaks of oxymethyl groups in PEG at *δ* 3.3 to 3.7 cover over the signals of pyranose ring of chitosan in the spectra. The weak and broad peaks at *δ* 4.3–4.5 are from the protons of -NH-CH (CH_2_)-CO- in TAT and MGF peptide. The multiple peaks at *δ* 6.5–8.5 belong to the MGF peptide sequence in CS-PEG-MGF polymer, and the peaks at *δ* 6.5–7.5 belong to the TAT peptide sequence in CS-PEG-TAT polymer. 

**Figure 2:**
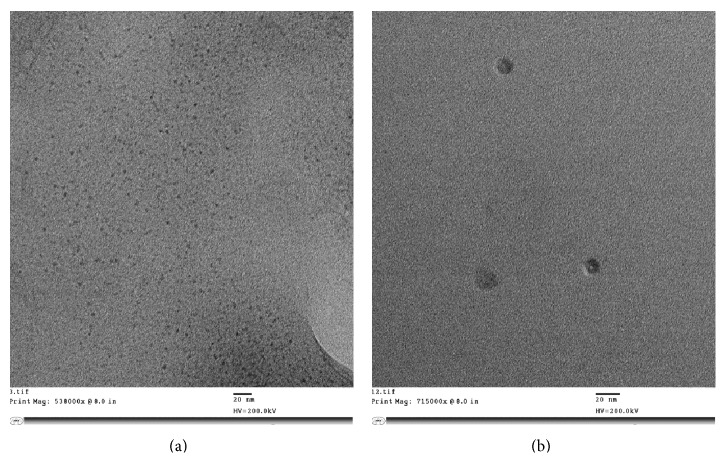
TEM study of TAT/MGF tagged PEGylated chitosan nanoparticles with siRNA magnification: (a) 538000x and (b) 715000x.

The nanoparticles were prepared following an ionic gelation scheme, as described previously by our group, wherein the positively charged polymer complexes with the negatively charged molecule (siRNA) due to the electrostatic interaction [35].  Figure 2 represents TEM images of nanoparticles prepared from CS-PEG-TAT/MGF nanoparticles at magnifications (a) 538000x and (b) 715000x, complexing siRNA at nitrogen : phosphate (N : P) ratio of 103.3 : 1 as previously optimized by our group [35]. The nanoparticles developed ranged from 5–10 nm in size and appeared spherical in shape, as observed under TEM.

### 3.2. Dose Optimization of siRNA/Nanoparticle Formulation to Target Brain Tissue In-Vivo

**Figure 3:**
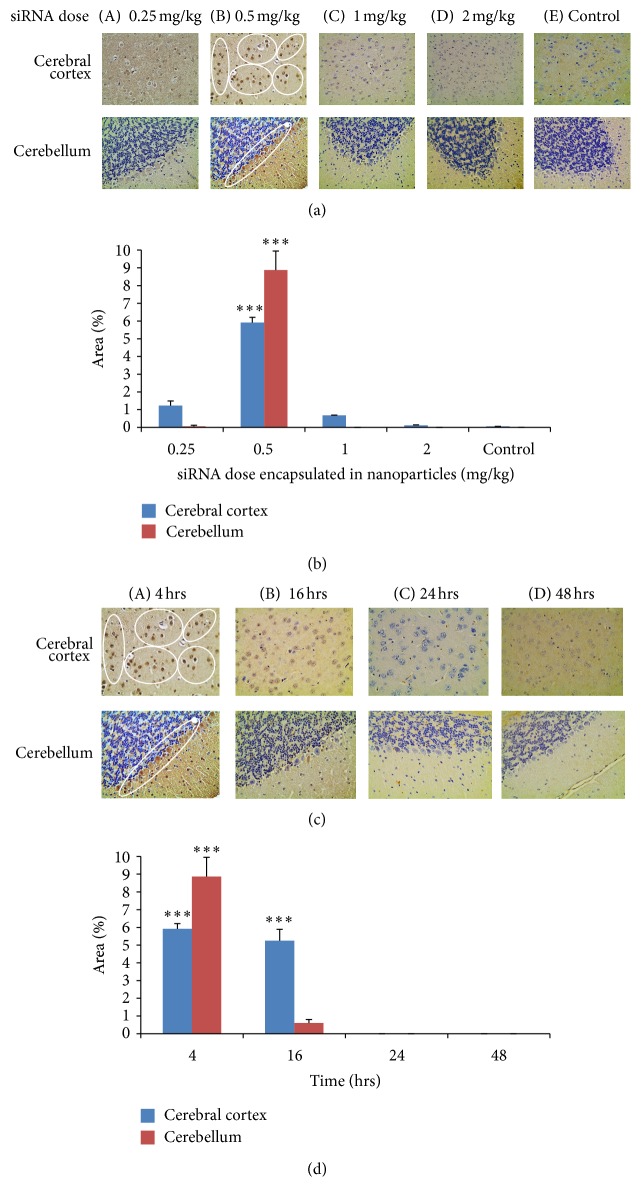
(a) Histopathological images of brain tissue (cerebral cortex and cerebellum) 4 hrs after receiving the nanoparticle formulation carrying doses of biotin-siRNA: (A) 0.25 mg/kg, (B) 0.5 mg/kg, (C) 1 mg/kg, (D) 2 mg/kg, and (E) control. (b) Quantitative analysis of the stained area in tissues using Image J. This study proved that the novel nanoparticle formulation successfully delivered the biotin-siRNA with high efficiency and selective targeting. The optimal dose of siRNA delivered via nanoparticles was determined to be 0.5 mg/kg. (c) Histopathological images of the cerebral cortex and cerebellum with nanoparticles carrying 0.5 mg/kg of biotin-siRNA at different time points, (A) 4 hrs, (B) 16 hrs, (C) 24 hrs, and (D) 48 hrs. (d) Quantitative analysis of the stained area in tissues using Image J. This study confirmed successful delivery of biotin-siRNA to the brain within 4 hrs of intranasal administration, with its clearance after 16 h. The graph shows a representative result of independent readings from two animals in each group (*n* = 2) mean ± s.d. ∗∗∗*P* < 0.01 was considered highly significant based on Tukey's post hoc analysis, when compared with other groups.

The optimal dose to be delivered to the four-week old C57BL/6J male mice was determined by administering the animals with different doses of biotin-tagged scrambled siRNA.  Figure 3(a) represents histopathological sections of the cerebral cortex and cerebellum from animals receiving different concentrations of nanoparticle-siRNA formulations. The animals were sacrificed after 4 h. The dark brown stained pyramidal neuronal cells obtained with 0.5 mg/kg of scrambled biotin-siRNA complexed nanoparticles ensured the delivery of siRNA in the neuronal cells of cerebral cortex (*P* = 0.0001) and in the Purkinje cells of cerebellum (*P* = 0.0001) as compared to the untreated control. Other animals that received 0.25 mg/kg of scrambled biotin-siRNA showed faint staining in the neuronal cells of cerebral cortex (*P* = 0.006); whereas, animals that received 1 and 2 mg/kg of scrambled biotin-siRNA dose did not show any staining in the tissue.  Figure 3(b) represents the quantitative analysis of the tissues using Image J software (NIH, USA), which calculates the mean percentage area of the dark brown stained cells.

The histopathological sections of the cerebral cortex and cerebellum at 0.5 mg/kg (determined as the optimal dose) at (A) 4, (B) 16, (C) 24, and (D) 48 h, as represented in Figure 3(c), show significant dark brown staining in the pyramidal neurons of the cerebral cortex and Purkinje cells of the cerebellum (*P* = 0.0001) at 4 hrs. The staining was observed only until 16 h in the cerebral cortex (*P* = 0.0001) and faded thereof, with no staining observed at 24 and 48 h. The result was quantified using Image J as represented in Figure 3(d). This study revealed that the delivery of scrambled biotin-siRNA by the TAT/MGF peptide-tagged PEGylated chitosan nanoparticles was achieved within 4 hrs of intranasal administration and was cleared after 16 h of administration. 

### 3.3. Biodistribution of siRNA/Nanoparticle Formulation In-Vivo

**Figure 4:**
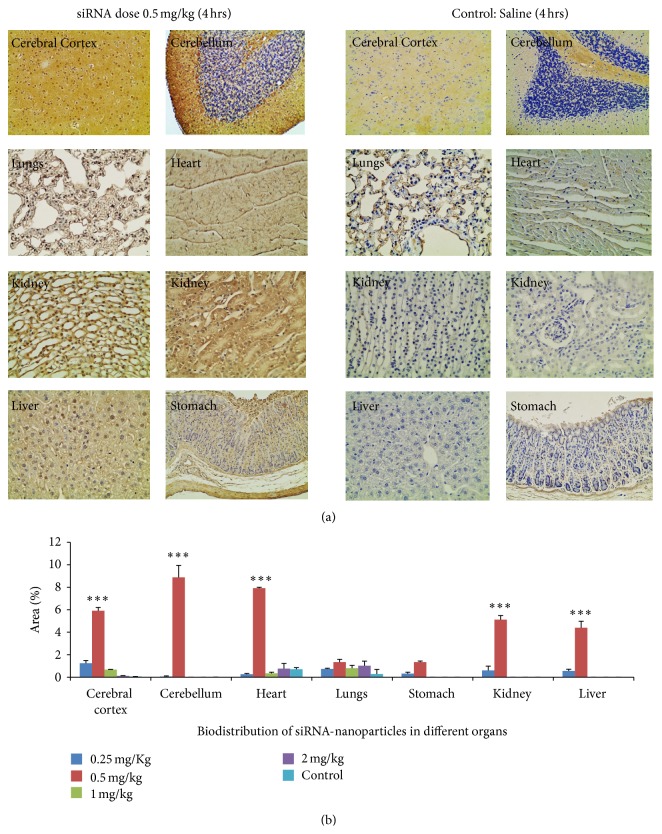
(a) Histopathological images of organ tissues collected 4 h following administration of the novel nanoparticle formulation containing biotin-siRNA dose at 0.5 mg/kg in animals, indicating biodistribution. The study confirmed maximum delivery of biotin-siRNA in the brain (cerebral cortex and cerebellum), with a lesser extent in the heart, kidney, liver, lungs, and stomach. The results of nanoparticle-based siRNA delivery on the left were compared to the untreated control animals on the right. (b) Quantitative analysis using Image J of the stained area in tissues from animals receiving scrambled biotin-siRNA dose, complexed in nanoparticles at 0.25 mg/kg, 0.5 mg/kg, 1 mg/kg, and 2 mg/kg, compared with the untreated control receiving 0.85% w/v NaCl. The graph shows a representative result of independent readings from two animals in each group (*n* = 2) mean ± s.d. ∗∗∗*P* < 0.01 was considered highly significant based on Tukey's post hoc analysis, when compared with other groups.

The biodistribution study was performed with animals receiving different concentrations of the biotin-siRNA/nanoparticle dose. The maximum biodistribution to other organs including brain was observed in animals that received 0.5 mg/kg of siRNA/nanoparticle dose.  Figure 4(a) represents histopathological sections of tissues from different organs receiving 0.5 mg/kg of biotin-siRNA/nanoparticle dose (left column) compared to untreated control (right column), sacrificed after 4 hrs of dose administration. The staining in the brain tissue was highly significant with 0.5 mg/kg scrambled biotin-siRNA/nanoparticle dose in both cerebral cortex and cerebellum (*P* = 0.0001) and also with 0.25 mg/kg but only in the cerebral cortex (*P* = 0.006), as represented in Figure 4(b). The staining with biotin-siRNA/nanoparticle dose at 0.5 mg/kg was also observed to target heart sarcomeres (*P* < 0.01) with significance as compared to other dose concentrations, 0.25 mg/kg (*P* = 0.403), 1 mg/kg (*P* = 0.562), and 2 mg/kg (*P* = 0.999) (Figure 4(b)). Renal cells in the medulla region of the kidney and hepatic cells also showed brown-colored staining in the cells, with 0.5 mg/kg of scrambled biotin-siRNA/nanoparticle formulation (*P* = 0.0001), as compared to the untreated control (Figure 4(b)). The glandular cells of the stomach and alveoli in lungs showed no significant difference as compared with the untreated control. Among all the concentrations of different treatment doses tested, the highest staining was observed with 0.5 mg/kg of scrambled biotin-siRNA/nanoparticle formulation in the cerebral cortex and cerebellum (*P* < 0.01), when compared with staining in other organs, except the heart.

### 3.4. Toxicity of siRNA/Nanoparticle Formulation In-Vivo

**Figure 5:**
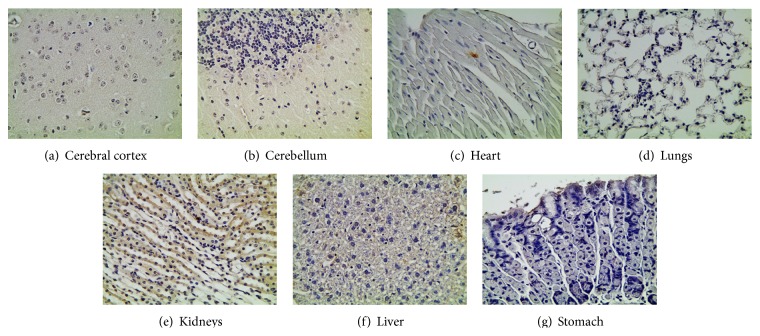
Histopathological images of various organ tissues collected 4 h following intranasal administration of multifunctional siRNA/nanoparticle formulation containing biotin-siRNA at a dose of 0.5 mg/kg. The tissues were stained with TUNEL-cell apoptosis assay. As indicated in the images, no apparent cell toxicity/apoptosis was observed in these tissues. This study confirms that the novel peptide-tagged nanoparticles were safe and did not cause any toxic effects.

Toxicity analysis was performed on the animals that received 0.5 mg/kg of the nanoparticles containing scrambled biotin-siRNA dose and were euthanized after 4 h, as represented in Figure 5. The tissue sections of various organs, such as brain, heart, lungs, kidney, liver, and stomach, were stained with TUNEL assay. The assay helps locate DNA damage in the cells resulting from apoptotic signaling cascades. The TUNEL assay was first modified to block the siRNA biotin tag to avoid any false positive results. Our results indicate that the scrambled biotin-siRNA/nanoparticle formulation had no toxicity/apoptotic effect, as no brown staining was detected in any of the tissues from different organs. 

## 4. Discussion

The research presented here demonstrates that the use of a surface functionalized chitosan nanoparticle formulation is capable of delivering siRNA to the brain, intranasally. The nanoparticles were developed using a novel synthetic scheme, comprising a parent polymer, chitosan, a hydrophilic polymer, PEG, and two peptides, TAT and MGF peptide. The modified polymer CS-PEG-TAT/MGF was used to form nanoparticles complexing siRNA following a previously established protocol by our group, published elsewhere [34]. In that study, the modified polymer synthesized was characterized by ^1^H NMR and Fourier transform infrared (FT-IR) spectroscopy at each intermediate step, and the nanoparticles developed, complexing siRNA at an N : P ratio of 103.3 : 1, were tested in-vitro on mouse neuroblastoma cells (Neuro2a) for transfection ability and cytotoxicity [34]. The results indicated efficient intracellular delivery of siRNA delivery, with minimal/no cytotoxicity [34]. The current study extends the potential of these developed nanoparticles to be used in-vivo. Apart from the biodegradable and biocompatible properties of chitosan polymer, its use as a parent polymer was preferred due to its cationic nature and the availability of functional groups that could be utilized to synthesize a surface graphed polymer. Moreover, the mucoadhesive character of the chitosan was an advantage for intranasal delivery. Considering the pH of nasal mucosa is 5.5–6.5 [36], the application of nanoparticle formulation at pH 5.5 was favored. In this study, the nanoparticles obtained were 5–10 nm in size as observed under TEM (Figure 2). Based on the mucoadhesive property of chitosan, a recent study showed intranasal application of chitosan adjuvanted influenza H5N1 vaccine to elicit mucosal and systemic immune responses [37]. The intranasal delivery is achieved by the absorption of the formulation across the nasal epithelia tissue, following the olfactory/trigeminal neural pathways [38]. This route bypasses the BBB and in addition evades the hepatic removal, glomerular filtration, and serum degradation of the nanoparticles [39, 40]. A review by Luppi et al. details the unique properties of chitosan and its applications for intranasal drug delivery [41].

PEG was utilized in the synthesis as a linker between chitosan and the peptide. PEG is a hydrophilic polymer, which reduces the toxicity of the nanoparticle and protects the payload from degrading enzymes [42]. The incorporation of TAT peptide provided a moiety for cell penetration [43]. Though it is nonselective in its mode of targeting, it has shown to permeate BBB in-vitro [44] and in-vivo [45]. Thus, it was used as a model peptide to enhance the permeation of nanoparticles through the BBB. The MGF peptide was used for its affinity towards neuronal tissues [46]. MGF is an alternatively spliced variant of insulin-like growth factor-1 (IGF-1) and has shown a neuroprotective effect in-vivo [47] and in-vitro, with its function being independent of IGF-1 receptor [48]. Thus, it is proposed that MGF has a different mode of action in terms of targeting neuronal tissues and neuroprotection [49]. 

The current study qualitatively investigates the potential of peptide-tagged PEGylated chitosan nanoparticles to deliver a scrambled biotin-siRNA to the brain (cerebral cortex and cerebellum) via intranasal route of administration, in-vivo. The results as observed in Figure 3(a), indicate that an optimal siRNA dose of 0.5 mg/kg was delivered through nanoparticles in the neuronal cells of the cerebral cortex and the Purkinje cells of the cerebellum. The presence of siRNA in the tissues was quantified by analysing the intensity of the brown-colored stained cells, indicating delivery of biotin-siRNA. The absence of stained cells at higher concentration, that is, 1 and 2 mg/kg of siRNA, was due to the clumping and aggregation of the siRNA-nanoparticle solution, when concentrated down to 30 *μ*L as a dose for intranasal application. The study also determined the pharmacokinetics of the siRNA/nanoparticle formulation and observed that the highest staining in the cells was observed at 4 h time point and was sustained until 16 h in the cerebral cortex (Figure 3(c)). No staining was observed in any tissue sections at the 24 h and 48 h time points. This reveals that the siRNA was delivered, and the nanoparticles were cleared from the system after 16 h of dose administration.

The current study also evaluated the site-specific delivery of the nanoparticles to the brain tissue by performing biodistribution analysis. As observed in Figure 3(c), the neurons in the cerebral cortex and the Purkinje cells of the cerebellum were intensely stained brown in color. However, a fair amount of staining was also observed in the heart tissue. The staining in the heart tissue is attributed to the small size of the nanoparticles of 5–10 nm that excavates into the systemic route and gets accumulated in other organs. The staining could also be attributed to the use of targeting peptide, MGF, which has also shown to have an affinity towards heart sarcomeres [50]. However, to confirm this statement, further studies would be required to test the biodistribution of siRNA delivered through chitosan-PEG without a targeting moiety, delivered the same way. The nanoparticles were also observed to be accumulated in the kidney and liver (Figure 4(b)), which is again attributed to the small size of the nanoparticles that leaked into the systemic route and were captured by the reticuloendothelial system and underwent hepatic filtration. A slight peripheral staining in the lungs and stomach was also observed. The toxicity analysis performed on the tissues of animals that received 0.5 mg/kg of siRNA dose in peptide-tagged PEGylated chitosan nanoparticles showed no apparent toxicity at the tissue level (Figure 5). 

Nanoparticle-mediated intranasal siRNA delivery has also been shown by other research groups. A study by Kim et al. showed intranasal delivery of siRNA against alpha B-crystallin, using a commercially available transfection reagent and gene knockdown in olfactory bulb, amygdala, and hypothalamus after 12 h of delivery [51]. Another study by the same research group showed intranasal siRNA delivery against HMGB1 gene, using PAMAM dendrimer formulation to cause neuroprotection in the postischemic brains of Sprague-Dawley rats [52]. Another study showed the intranasal delivery of radioactive ^32^P-siRNA dendriplexes complexed in mucoadhesive gels with maximum expression in the olfactory bulb [23]. These applications are in accordance to the study performed by our group, suggesting intranasal delivery is a well established route to deliver nanoparticle formulations targeting brain and causing a therapeutic effect. Though, researchers have explored the intranasal route of delivery with various nanoparticle formulations, this study is unique in representing the delivery of peptide-tagged nanoparticle formulation to deliver siRNA specifically in the brain tissue. The incorporation of model peptides, TAT and MGF, in the synthesis proved that the formulation is multifunctional. The advantage of this formulation is its ability to be tailor-made in terms of application. The peptides, TAT and MGF, which were used as model ligands can be replaced with an antibody or a ligand specific for the cell surface receptor. The use of other peptides and growth factors in conjunction with a delivery vehicle, carrying a therapeutic, can provide a noninvasive solution to neurological disorders. The current study presented is the proof-of-concept, which determined the delivery aspect of the nanoparticle formulation. However, further studies would be needed to evaluate the biological efficacy of the intranasal route for delivery and to direct the application of the developed nanoparticles towards a specific neurological condition. This would involve (1) delivery of a functional siRNA targeting specific neurons involved in the diseased condition to cause a therapeutic effect, (2) the utilization of a specific targeting ligand towards the cell-surface receptor, and (3) evaluation of targeting efficacy with nontargeting nanoparticles and siRNA delivered without a delivery vehicle as controls. Our further studies would involve the consideration of above parameters to prove the efficacy of the delivery formulation in an animal model of neurodegenerative disease. 

## 5. Conclusion 

The current study evaluated the optimal dose for the delivery of siRNA using surface-functionalized, peptide-tagged PEGylated chitosan nanoparticles to the cerebral cortex and cerebellum via intranasal route. The nanoparticles developed were 5–10 nm in size carrying siRNA at an N : P ratio of 103.3 : 1. The siRNA dose determined for an optimal delivery, targeting neuronal cells, was 0.5 mg/kg. The siRNA delivery was found to be significant in cerebral cortex and cerebellum after 4 hrs of intranasal postdelivery. Furthermore, the biodistribution and toxicity characterization demonstrated maximum siRNA delivery in the brain with no visible, local toxic effects linked to delivery of the nanoparticle formulation at cellular levels. Hence, the developed MGF/TAT tagged PEGylated chitosan/siRNA nanoparticle formulation shows a great promise for use as a therapeutic modality in the treatment and prevention of neurodegenerative disorders. However, further studies would be performed to evaluate the efficacy of these nanoparticles to specifically target the diseased cells, in-vivo.
